# Metabolomic and Lipidomic Profiling of Preoperative CSF in Elderly Hip Fracture Patients With Postoperative Delirium

**DOI:** 10.3389/fnagi.2020.570210

**Published:** 2020-10-22

**Authors:** Yongzheng Han, Wenchao Zhang, Jiao Liu, Yanan Song, Taotao Liu, Zhengqian Li, Xiaoxiao Wang, Ning Yang, Yue Li, Dengyang Han, Xinning Mi, Yang Zhou, Min Li, Xiangyang Guo, Lijun Zhong, Geng Wang, Yi Yuan

**Affiliations:** ^1^Department of Anesthesiology, Peking University Third Hospital, Beijing, China; ^2^Department of Anesthesiology, Beijing Jishuitan Hospital, Beijing, China; ^3^Center of Medical and Health Analysis, Peking University Health Science Center, Beijing, China; ^4^Research Center of Clinical Epidemiology, Peking University Third Hospital, Beijing, China

**Keywords:** cerebrospinal fluid, postoperative delirium, hip fracture, metabolomics, lipidomics

## Abstract

**Objective:**

To investigate dysregulated molecules in preoperative cerebrospinal fluid (CSF) of elderly hip fracture patients with postoperative delirium (POD), in order to identify potential pathological mechanisms and biomarkers for pre-stage POD.

**Materials and Methods:**

This nested case control study used untargeted metabolomic and lipidomic analysis to profile the preoperative CSF of patients (*n* = 40) who developed POD undergone hip fracture surgery (*n* = 10) and those who did not (*n* = 30). Thirty Non-POD patients were matched to 10 POD patients by age (± 2 years) and Mini Mental State Examination score (± 2 points). CSF was collected after successful spinal anesthesia and banked for subsequent analysis. On the first two postoperative days, patients were assessed twice daily using the Confusion Assessment Method-Chinese Revision. CSF samples from the two groups were analyzed to investigate possible relevant pathological mechanisms and identify candidate biomarkers.

**Results:**

Demographic characteristics of the groups were matched. Eighteen metabolites and thirty-three lipids were dysregulated in the preoperative CSF of POD patients. Pathway enrichment analysis revealed perturbations in D-glutamine and D-glutamate metabolism; glycerophospholipid metabolism; alanine, aspartate and glutamate metabolism; sphingolipid metabolism; histidine metabolism; and arginine biosynthesis at the pre-delirium stage. Receiver operating characteristic curve analysis indicated that phosphatidylethanolamine (PE, 40:7e), with an area under the curve value of 0.92, is a potential biomarker for POD.

**Conclusion:**

Multiple pathological mechanisms in the POD group were involved before surgery, including neuroinflammation, oxidative stress, and energy metabolism disorders induced by hypoxia, as well as neurotransmitter imbalances such as increased dopamine and glutamate, and decreased glutamine. These metabolic abnormalities potentially increase the fragility of the brain, thus contributing to POD. PE (40:7e) might be a potential biomarker for POD. Not only do our results provide potential biomarkers for POD, but also provide information for deep pathological research.

**Clinical Trial Registration:**

www.ClinicalTrials.gov, identifier ChiCTR1900021533.

## Introduction

Patients over 65 years of age are the largest consumers of procedural care, and postoperative delirium (POD) is one of the most common complications experienced by elderly patients during the postoperative period (Daiello et al., 2019). POD is an acute neuropsychiatric syndrome occurring in the hours to days after anesthesia and surgery (Vutskits and Xie, 2016), which can elicit durable deficits in executive function, memory, attention, and other cognitive domains (Eckenhoff et al., 2020). The incidence of POD is 20–45% among elderly adult surgery patients (Rudolph and Marcantonio, 2011; Inouye et al., 2014). As for hip fracture induced by traumatic stimulation in elderly patients, the incidence may reach as high as 53.3% (Aldecoa et al., 2017). POD primarily occurs 24–72 h after surgery, and most symptoms disappear in 1 week. However, it is linked with persistent impairments in brain function, including cognitive decline (Inouye et al., 2016), increased risk for Alzheimer’s disease (AD) (Olofsson et al., 2018), and serious negative outcomes on patient prognosis such as longer hospitalization, decline in physical function, and even death (Schmitt et al., 2012). Considering the aggravation of an aging global population, the incidence of POD has become a major evaluation index of medical quality and safety (Berian et al., 2018).

As for POD patients, their preoperative brain functional reserve has already decreased, therefore, they have a higher risk to develop delirium after surgery. Thus, screening and providing greater attention to these potential higher-risk patients represents an important issue. The blood–brain barrier, which acts as a strict control point for what can enter the brain, is made up of tight junctions between endothelial cells lining blood vessels, astrocytic end feet, and a basement membrane. As an overwhelming majority of molecules indicative of central nervous system damage cannot be detected in the vasculature, cerebrospinal fluid (CSF) is the best body fluid to accurately demonstrate and evaluate biochemical changes following brain damage. Despite the prevalence and clinical significance of POD, its pathophysiology is still unclear. Clinical symptoms fluctuate and, at present, no reliable biomarkers have been identified. In view of this, identifying potential indicators for POD is an urgent clinical task. Evaluating dysfunctional metabolite expression in CSF using metabolomic and lipidomic analysis may enhance our understanding of pathological changes in POD patients at the molecular level.

We hypothesized that differentially expressed metabolites and lipids in preoperative CSF are associated with POD in elderly orthopedic patients. Our findings provide valuable scientific clues for the investigation of POD neuropathogenesis and facilitate more specific biomarker studies in the field.

## Materials and Methods

### Patients and Setting

**FIGURE 1:**
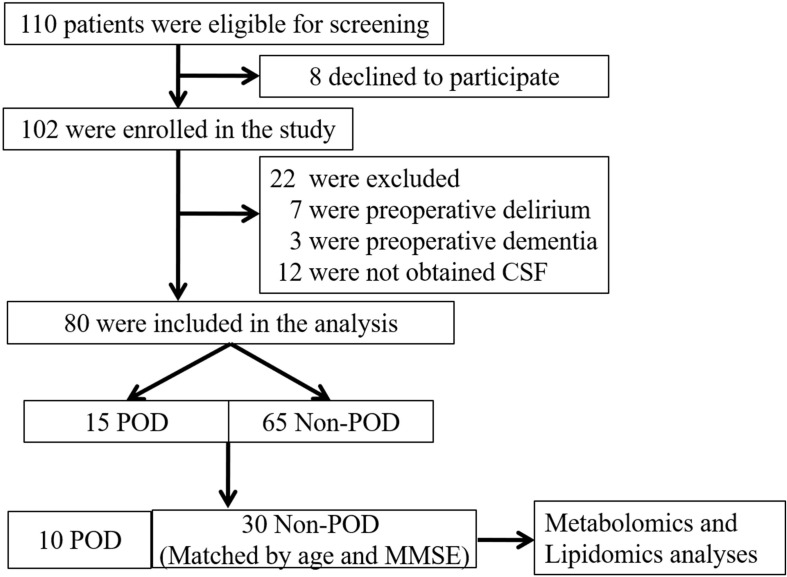
Flow diagram showing the recruiting criterion. One hundred and ten patients were initially screened for the study, and 40 patients were finally included in the data analysis. CSF, cerebrospinal fluid; POD, postoperative delirium; MMSE, mini-mental state examination.

This study was approved by the Beijing Jishuitan Hospital Medical Science Research Ethics Committee (JLKS201901-04) and conformed to the principles of the Declaration of Helsinki. CSF samples were obtained for the purpose of laboratory research. All methods were performed in accordance with relevant guidelines and regulations. With written informed consent, this study was registered at the Chinese Clinical Trial Registry (ChiCTR1900021533). All participants were recruited from Beijing Jishuitan Hospital (Beijing, China) from March 2019 to August 2019. Eligible patients were at least 65 years of age with acute hip fracture injury (no longer than 72 h) and scheduled for hip internal fixation or hip arthroplasty by the same surgical team under spinal anesthesia. Postoperatively, all patients received intravenous patient-controlled analgesia with the same regimen (100 μg sufentanil and 8 mg ondansetron in 100 mL normal saline). A total of 110 adults were recruited in this study (Figure 1). Patients were excluded if they: (Daiello et al., 2019) had a past medical history of neurological disease such as delirium, schizophrenia, dementia, or stroke [with regard to dementia, it was screened if Mini Mental State Examination (MMSE) scores of ≤ 17 for illiterate patients, ≤ 20 for patients with 1–6 years of education, or ≤ 24 for patients with 7 or more years of education] (Li et al., 2016; Vutskits and Xie, 2016) were unable to read or write or cooperate; (Eckenhoff et al., 2020) had a history of drug or alcohol abuse. Eighty participants (15 POD vs. 65 Non-POD) completed the study. In the 15 POD patients, 5 POD patients’ CSF were mixed with blood during the spinal anesthesia. In order to rule out the influence of blood on the results of CSF metabolomics and lipidomics to the greatest extent, we finally chose to analyze the 10 POD patients and the matched 30 Non-POD patients by age (± 2 years) and MMSE score (± 2 points), to make sure the two groups of patients are comparable. After admission, all patients were administered oxycodone/acetaminophen (5/325 mg, four times a day) to relieve pain.

### Dietary Management

After admission, all patients were placed on a bland diet and began fasting at 22:00 on the night before surgery. Patients were started on a semiliquid diet 6 h after surgery and then subsequently advanced to a bland normal diet.

### Anesthesia and Surgery

All participants underwent hip internal fixation or hip arthroplasty under spinal anesthesia. All surgeries were performed by the same surgical team to avoid potential confounding factors caused by varying surgical skills or different surgical practices. Electrocardiography, pulse oximetry, and non-invasive blood pressure were all continuously monitored during anesthesia and were recorded at 3 min fixed intervals. Postoperatively, all patients received intravenous patient-controlled analgesia with the same regimen (100 μg sufentanil and 8 mg ondansetron in 100 mL normal saline).

### Neuropsychological Testing

We interviewed all patients the day before surgery and performed MMSE. The Confusion Assessment Method (CAM) was used to identify patients who experienced preoperative delirium. CAM was performed twice daily on the first and second days after surgery (8:00 and 20:00). According to a previous study, POD was usually diagnosed in elderly hip fracture surgery patients during this period (Scholtens et al., 2016). The presence or absence of POD was defined according to the results of the Chinese version of CAM, which has good reliability and validity in the elderly Chinese population (Shi et al., 2014).

### CSF Sample Preparation

After successful administration of spinal anesthesia, but prior to the administration of local anesthetic, CSF (2 mL) was collected in a polypropylene tube and placed on ice. Samples were immediately centrifuged at 3,000 × g for 10 min at 4°C to remove cells (Muller et al., 2014), and the supernatant was aliquoted and stored at –80°C until analysis. Metabolites and lipids were extracted from CSF samples using liquid–liquid extraction. Briefly, 500 μL of CSF were concentrated to 50 μL and mixed with a fourfold volume of ice-cold chloroform/methanol (2:1, v/v). After vortexing for 15 min at 4°C, the mixture was centrifuged at 13,000 × g for 15 min. The upper aqueous phase (hydrophilic metabolites) and lower organic phase (hydrophobic metabolites) were separately collected and evaporated at room temperature under vacuum. All evaporated samples were stored at −80°C until high-performance liquid chromatography–mass spectrometry (HPLC–MS) analysis.

### High-Performance Liquid Chromatography

Metabolomics and lipidomics were performed on an Ultimate 3000 UHPLC system coupled with a Q-Exactive HF MS (Thermo Scientific, Waltham, MA). For the aqueous phase (metabolomics), an Xbridge amide column (100 × 2.1 mm i.d., 3.5 μm; Waters Corporation, Milford, MA) was employed for compound separation at 30°C. Samples were suspended in 100 μL of acetonitrile:water (1:1, v/v), and the injection volume was 10 μL. Mobile phase A consisted of 5 mM ammonium acetate in water with 5% acetonitrile, and mobile phase B was acetonitrile. The flow rate was 0.4 mL/min with the following linear gradient: 0 min, 95% B; 3 min, 90% B; 13 min, 50% B, 14 min, 50% B; 15 min, 95% B; and 17 min, 95% B.

As for lipids, chromatographic separation was performed on a reverse-phase X-select CSH C18 column (2.1 × 100 mm, 2.5 μm; Waters) at 40°C. Two solvents, both containing 10 mM ammonium acetate and 0.1% formic acid, were used for gradient elution: (A) ACN/water (3:2, v/v) and (B) IPA/ACN (9:1, v/v). The gradient program was: 0 min, 40% B; 2 min, 43% B; 12 min, 60% B; 12.1 min, 75% B; 18 min, 99% B; 19 min, 99% B; and 20 min, 40% B. The flow rate was set to 0.4 mL/min. Samples were suspended with 100 μL of chloroform:methanol (1:1, v/v) and diluted threefold with isopropanol:acetonitrile:H_2_O (2:1:1, v/v/v). The injection volume was 10 μL.

### Mass Spectrometry

Data-dependent acquisition (DDA) was performed using Q-Exactive HF MS (Thermo Scientific). For DDA-MS, acquisition was performed in the positive and negative switching ion mode under profile type. Each acquisition cycle consisted of 1 survey scan (MS1 scan) at 60,000 resolution from 60 to 900 m/z for hydrophilic metabolites and mass range m/z 300 to 1,200 for lipids, followed by 10 MS/MS scans in HCD mode at 30,000 resolution using stepped normalized collision energies of 15, 30, and 45. Dynamic exclusion was set to 8 s. The automatic gain control target was set to 5e6 (30 ms maximum injection time) and 2e5 (100 ms maximum injection time) for MS1 and MS/MS scans, respectively. Parameters of the ion source were: spray voltage of 3.3 kV for positive ion mode and 3.0 kV for negative ion mode; ion source sheath gas = 40; auxiliary gas = 10; capillary temperature = 320°C; probe heater temperature = 300°C; and S-lens RF level = 55. Samples were analyzed in random order. Quality control (QC) samples were prepared by pooling equal volumes of all study instances, and were analyzed between every five samples during the entire LC–MS analytical sequence.

### DDA-MS Data Analysis

Raw data collected from DDA–MS were processed on MS–DIAL software (Tsugawa et al., 2015) according to the user guide. Briefly, raw MS data were converted from the vendor file format (.wiff) into the common file format of Reifycs Inc. (.abf) using the Reifycs ABF converter^1^. After conversion, MS–DIAL software was used for feature detection, spectra deconvolution, metabolite identification, and peak alignment between samples. MS/MS spectra-based metabolite identification was performed in MS–DIAL by searching the acquired MS/MS spectra against the MassBank database. It contains MS1 and MS/MS information of metabolites (3,928 records in positive ion mode and 4,963 records in negative ion mode). MS/MS spectra-based lipid identification was performed in MS–DIAL by searching the acquired MS/MS spectra against the internal *in silico* MS/MS spectra database. It includes MS1 and MS/MS information of common lipid species. Tolerances for MS1 and MS/MS searches were set to 0.01 and 0.05 Da, respectively. Other MS–DIAL parameters were set to default values.

### Statistical Analysis

Principal component analysis (PCA) and partial least squares discriminant analysis (PLS-DA) were implemented to visualize the quality of metabolic profiling and metabolic differences between POD and Non-POD groups. Significantly altered metabolites with variable importance in projection (VIP) values >1 in the abovementioned PLS-DA model, as well as differing *p*-values determined by Student’s *t*-test (*p* < 0.05), were selected in POD and Non-POD groups. PCA, PLS-DA, and pathway enrichment analysis for DDA data were performed with Metaboanalyst 4.0^2^, an online tool for analyzing omics data. Furthermore, the Kyoto Encyclopedia of Genes and Genomes (KEGG) database was used to identify pathways associated with altered metabolites.

SPSS software (version 21.0; IBM Corporation, Armonk, NY) was used for data analysis. Data are expressed as mean ± SD, median and interquartile range (IQR), or number (%). The Kolmogorov–Smirnov method was used to test the normality of all variables. Categorical variables were analyzed using a χ^2^-test, while continuous variables were analyzed using an independent-samples *t*-test. The Mann–Whitney *U*-test was used to analyze non-normal variables. Statistical significance was set at *p* < 0.05. Given the success of PLS-DA models in classifying POD and Non-POD groups, the top 20 differentially expressed lipids according to VIP were identified. Afterward, receiver operating characteristic (ROC) curves for dysregulated molecules were calculated in order to evaluate their potential use as candidate biomarkers for POD; the area under the curve (AUC) provides a global summary statistic of test accuracy.

## Results

### Participant Characteristics

In this study, 10 CSF samples were collected from patients with CAM-confirmed POD, while 30 matched CSF samples were collected from patients without POD. There were no differences in age, MMSE score, gender, height, weight, body mass index, American Society of Anesthesiologists (ASA) physical class, education years, length of anesthesia and surgery, Charlson comorbidity score, or preoperative visual analog scale between POD and Non-POD groups (Table 1).

**TABLE 1:** Subject characteristics.

	**POD group (*n* = 10)**	**Non-POD group (*n* = 30)**	**Statistical test**	***P*-value**
Age (years), mean ± *SD*	82.2 ± 7.6	81.7 ± 7.2	*t* = 0.188	0.852
MMSE score, mean ± *SD*	25.2 ± 3.9	25.4 ± 3.6	*t* = -0.174	0.863
Male, *n* (%)	5 (50.0)	7 (23.3)	χ^2^ = 1.429	0.232
Height (cm), mean ± *SD*	165.0 ± 7.9	163.2 ± 9.4	*t* = 0.516	0.609
Weight (kg), mean ± *SD*	66.1 ± 14.2	63.1 ± 10.5	*t* = 0.647	0.522
BMI (kg/m^2^), mean ± *SD*	24.6 ± 5.4	23.3 ± 3.0	*t* = 0.846	0.404
ASA class, *n* (%)			χ^2^ = 0.342	0.559
II	8 (80.0)	19 (63.3)		
III	2 (20.0)	11 (36.7)		
Education (years), median (IRQ)	13.5 (7.8)	9.0 (9.8)	*z* = -0.793	0.428
Length of anesthesia (min), mean ± *SD*	91.9 ± 12.5	93.8 ± 30.7	*t* = -0.171	0.865
Length of surgery (min), mean ± *SD*	63.8 ± 11.6	75.8 ± 33.4	*t* = -0.994	0.328
Charlson comorbidity score, mean ± *SD*	5.9 ± 1.0	6.3 ± 1.7	*t* = -0.671	0.506
Preoperative VAS score, mean ± *SD*	3.6 ± 1.0	3.2 ± 0.9	*t* = 0.786	0.441

**POD, postoperative delirium; MMSE, Mini-Mental State Examination; ASA, American Society of Anesthesiologists; IQR, interquartile range; VAS, Visual Analogue Scale; cm, centimeter; kg, kilogram; min, minute; SD, standard deviation.**

### Untargeted Metabolic Profile Before Surgery

**FIGURE 2:**
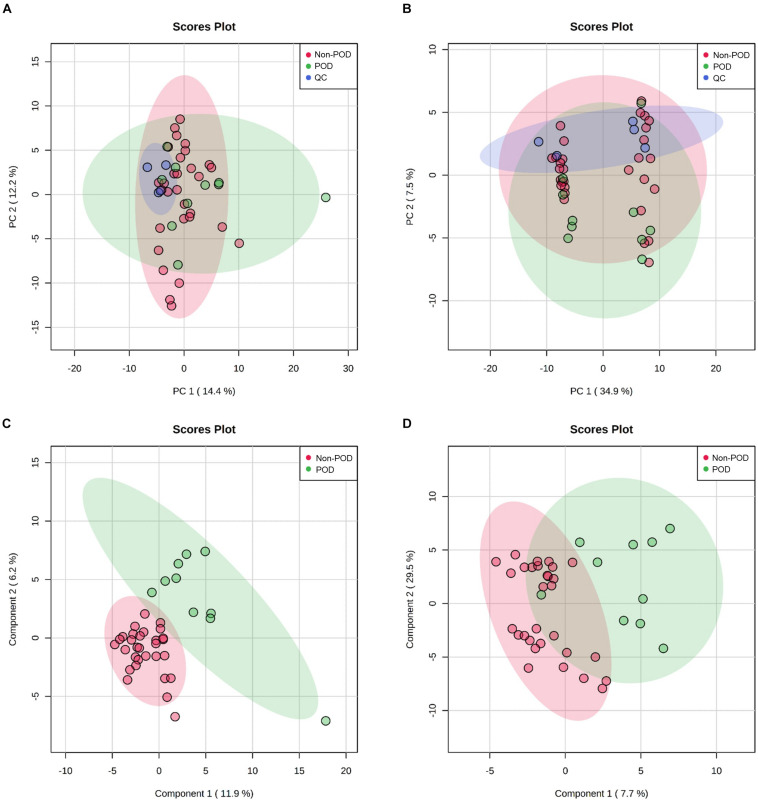
Untargeted metabolic profiling of CSF samples in POD patients and Non-POD patients. PCA (**A:** positive-ion mode; **B:** negative-ion mode) and PLS-DA (**C:** positive-ion mode; **D:** negative-ion mode) analyses of the DDA-based metabolomics data. The indicated groups are presented by different colors (green: POD; red: Non-POD; blue: QC).

After processing the raw MS data, PCA analysis was used to create an overview of metabolomic expression profiles of all samples in positive and negative ion modes. Pooled QC samples were clustered well in the PCA score plots. In addition, POD and Non-POD groups clustered successfully in the PLS-DA model (Figure 2). After peak alignment and the removal of missing values, 202 features in positive ion mode and 156 in negative ion mode were reliably identified by MS/MS spectra comparison. Of these features, further statistical analysis revealed a total of 18 dysregulated metabolites between POD and Non-POD groups (VIP > 1 and *t*-test *p* < 0.05, Table 2).

**TABLE 2:** Differentiating metabolites between POD and Non-POD groups identified from the metabolomic data.

**Pathway**	**Metabolites**	**VIP**	***P*-value**	**FC (P/N)**	**Trend**
Amino sugar metabolism	N-Acetylmannosamine	2.67	1.52E-03	0.39	Down
Amino acid metabolism	L-Saccharopine	2.60	1.88E-03	0.57	Down
Fatty acid metabolism	9-Trans-Palmitelaidic acid	2.53	2.64E-03	2.58	Up
Fatty acid metabolism	Citramalate	2.51	2.82E-03	0.66	Down
Glycolysis	D-Glucose 6-phosphate	2.23	8.85E-03	2.94	Up
Amino acid metabolism	Creatine	2.21	9.51E-03	0.75	Down
Histidine metabolism	Histamine	2.21	1.00E-02	0.47	Down
Amino acid metabolism	Methionine	2.21	1.01E-02	0.57	Down
Fatty acid metabolism	Trans-Vaccenic acid	2.2	1.02E-02	0.88	Down
Amino sugar metabolism	N-Acetylgalactosamine	2.14	1.28E-02	0.26	Down
Amino acid metabolism	Glutamate	2.14	1.32E-02	4.21	Up
Purine metabolism	Hypoxanthine	2.11	1.41E-02	0.67	Down
Amino acid metabolism	N-Methylproline	1.98	2.28E-02	0.47	Down
Amino acid metabolism	Glutamine	1.96	2.33E-02	0.64	Down
Lipid metabolism	rac-Glycerol 3-phosphoate	1.91	2.70E-02	0.60	Down
Lipid metabolism	sn-Glycero-3-phosphocholine	1.86	3.25E-02	1.54	Up
Amino sugar metabolism	D-Glucosamine-6-phosphate	1.79	4.02E-02	0.63	Down
Amino acid metabolism	Dopamine	1.71	4.94E-02	13.15	Up

**VIP, variable importance in the projection; FC, fold change.**

### Untargeted Lipidomic Profile Before Surgery

**FIGURE 3:**
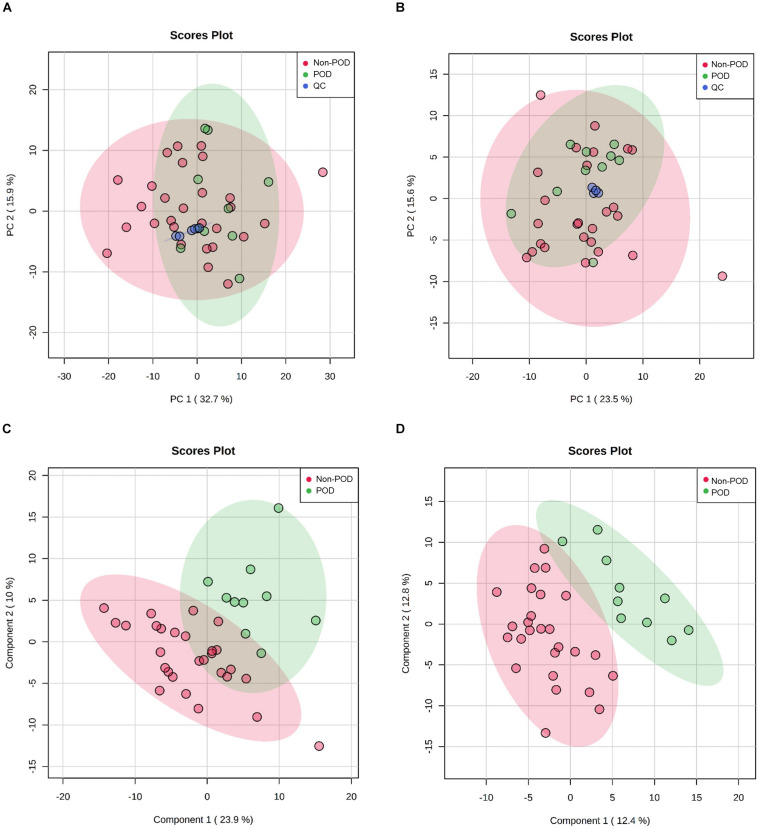
Untargeted lipidomics profiling of CSF samples in POD patients and Non-POD patients. PCA (**A:** positive-ion mode; **B:** negative-ion mode) and PLS-DA (**C:** positive-ion mode; **D:** negative-ion mode) analyses of the DDA-based lipidomics data. The indicated groups are presented by different colors (green: POD; red: Non-POD; blue: QC).

As with the data processing procedures for metabolomic features, PCA and PLS-DA were used to generate an overview of the expression patterns of lipids in all samples (Figure 3). Similar to the observed results of metabolomic profiles, lipid QC samples were closely gathered in the PCA score plots in both positive and negative ion modes. In total, 385 features in positive ion mode and 348 in negative ion mode were reliably identified by MS/MS spectra comparison. Statistical analysis revealed 33 lipids (Supplementary Table S1) were dysregulated between POD and Non-POD groups (VIP > 1 and *t*-test *p*-value < 0.05). The top 20 dysregulated lipids in POD patients according to VIP are shown in Table 3.

**TABLE 3:** The top 20 differentiating lipids between POD and Non-POD groups identified from the lipidomic data.

**Pathway**	**Lipids**	**VIP**	***P*-value**	**FC (P/N)**	**Trend**
Sphingolipid metabolism	Cer-NS (d40:2); Cer-NS (d18:1/22:1)	2.96	4.41E-05	2.51	Up
Glycerophospholipid metabolism	PE (40:6); PE (18:0–22:6)	2.86	8.00E-04	0.59	Down
Glycerophospholipid metabolism	PE (38:7e); PE (16:1e/22:6)	2.76	1.35E-03	0.66	Down
Glycerophospholipid metabolism	PE (40:7e); PE (18:1e/22:6)	2.70	1.74E-03	0.43	Down
Glycerolipid metabolism	DAG (44:5e); DAG (22:3e/22:2)	2.61	4.52E-04	0.47	Down
Glycerolipid metabolism	LDGCC (34:1)	2.49	9.05E-04	0.6	Down
Sphingolipid metabolism	Cer-NS (d50:1); Cer-NS (d22:1/28:0)	2.39	1.61E-03	2.18	Up
Sphingolipid metabolism	Cer-NS (d52:1); Cer-NS (d22:1/30:0)	2.27	2.89E-03	3.00	Up
Sphingolipid metabolism	Cer-NS (d42:4); Cer-NS (d22:3/20:1)	2.26	3.03E-03	1.79	Up
Glycerophospholipid metabolism	PC (34:3)	2.18	4.47E-03	75.52	Up
Glycerophospholipid metabolism	PC (40:6); PC (18:0–22:6)	2.12	1.69E-02	0.51	Down
Glycerophospholipid metabolism	PC (33:1); PC (16:0–17:1)	2.06	2.04E-02	0.74	Down
Glycerophospholipid metabolism	PC (32:2)	2.02	8.79E-03	1.60	Up
Sphingolipid metabolism	Sphinganine (25:0)	2.02	9.11E-03	0.42	Down
Sphingolipid metabolism	SM (d34:1); SM (d18:1/16:0)	1.96	1.17E-02	4.78	Up
Sphingolipid metabolism	SM (d44:2); SM (d21:2/23:0)	1.92	1.34E-02	1.59	Up
Sphingolipid metabolism	SM (d34:2); SM (d14:2/20:0)	1.84	1.84E-02	1.26	Up
Glycerophospholipid metabolism	PC (37:3)	1.82	1.99E-02	15.33	Up
Glycerophospholipid metabolism	PC (33:2)	1.80	2.14E-02	1.65	Up
Sphingolipid metabolism	SM (d42:2); SM (d18:1/24:1)	1.79	2.18E-02	1.27	Up

**VIP, variable importance in the projection; FC, fold change; Cer-NS, ceramide non-hydroxyfatty acid-sphingosine; PE, phosphatidylethanolamine; DAG, diacylglycerol; LDGCC, lysodiacylglyceryl-3-O-carboxyhydroxymethylcholine; PC, phosphatidylcholine; SM, sphingomyelin.**

### Bioinformatic Analysis Revealed Perturbed Metabolic Pathways

**FIGURE 4:**
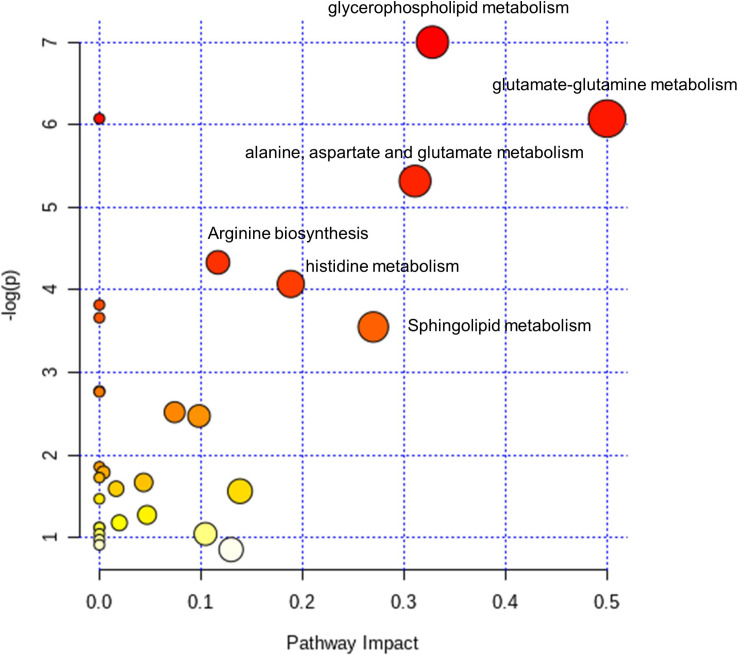
Pathway analysis of the dysregulated metabolites and lipids in the CSF of POD patients. Fifteen selected metabolites and lipids were involved in the D-Glutamine and D-glutamate metabolism (2 hit), Glycerophospholipid metabolism (4 hits), Alanine, aspartate and glutamate metabolism (3 hits), Sphingolipid metabolism (2 hits), Histidine metabolism (2 hits) and Arginine biosynthesis (2 hits). The color gradient indicates the significance of the pathway ranked by *p*-value (y-axis; yellow: higher *p*-values, red: lower *p*-values), and circle size indicates the pathway impact score (x-axis; the larger circle, the higher impact score). Significantly affected pathways with low *p*-values and high pathway impact scores are labeled.

The 18 dysregulated metabolites and 20 dysregulated lipids were subjected to KEGG pathway enrichment analyses. Further bioinformatic analyses were employed to reveal perturbed pathways and provide clues for the underlying pathological mechanism. According to pathway impact and a *p*-value of < 0.05, the top six metabolic pathways significantly perturbed in POD patients (Figure 4) were D-glutamine and D-glutamate metabolism (two hits: glutamate and glutamine); glycerophospholipid metabolism [nine metabolites in four hits: phosphatidylethanolamine (PE) (40:6), PE (38:7e), PE (40:7e), phosphatidylcholine (PC) (34:3), PC (40:6), PC (32:2), PC (33:2), sn-glycero-3-phosphocholine, and rac-glycerol 3-phosphoate]; alanine, aspartate, and glutamate metabolism (three hits: glutamate, glutamine, and D-Glucosamine-6-phosphate); sphingolipid metabolism [two hits: sphingomyelin (SM) (d34:1) and SM (d42:2)]; histidine metabolism (two hits: histamine and glutamate); and arginine biosynthesis (two hits: glutamate and glutamine).

### Metabolites Associated With Potential Biomarkers for POD

**FIGURE 5:**
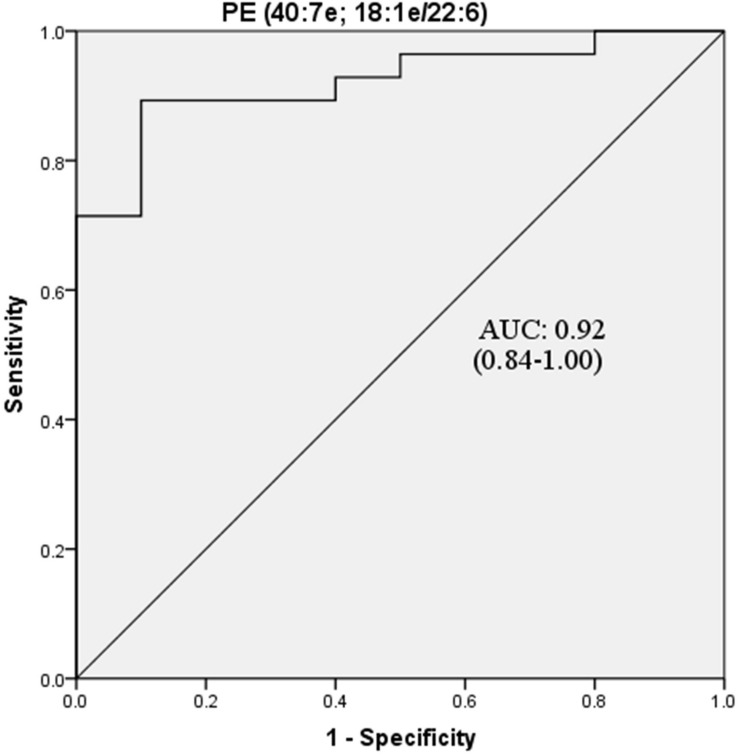
ROC curve analysis of potential CSF biomarker for differentiating the POD group from the Non-POD group. PE, phosphatidylethanolamine. The area under the curve for the prediction of POD via PE (40:7e) was 0.92 (95% CI = 0.84–1.00).

Further ROC analysis revealed four metabolites and eight lipids with an AUC greater than 0.8 (Table 4). In particular, PE (40:7e), which had the largest AUC value of 0.92, could be considered a potential biomarker for POD (Figure 5).

**TABLE 4:** AUCs for metabolites and lipids.

**Metabolites or lipids**	**AUC**	**95% CI**	***P* Value**	**SE**
PE (40:7e); PE (18:1e/22:6)	0.92	0.84–1.00	9.20E-05	0.04
Cer-NS (d40:2); Cer-NS (d18:1/22:1)	0.90	0.80–1.00	2.05E-04	0.05
DAG (44:5e); DAG (22:3e/22:2)	0.88	0.77–0.99	4.42E-04	0.06
PE (38:7e); PE (16:1e/22:6)	0.84	0.71–0.98	1.46E-03	0.07
PE (40:6); PE (18:0–22:6)	0.84	0.70–0.98	1.64E-03	0.07
L-Saccharopine	0.84	0.67–1.00	1.46E-03	0.09
PC (40:6); PC (18:0–22:6)	0.83	0.69–0.96	2.56E-03	0.07
N-Acetylmannosamine	0.83	0.67–0.98	2.56E-03	0.08
Sphinganine (25:0)	0.82	0.69–0.96	2.85E-03	0.07
9-Trans-Palmitelaidic acid	0.81	0.67–0.95	4.36E-03	0.07
Cer-NS (d42:4); Cer-NS (d22:3/20:1)	0.80	0.65–0.96	4.84E-03	0.08
Citramalic acid	0.80	0.63–0.98	4.84E-03	0.09

**AUC, area under the curve; 95% CI, 95% confidence interval; SE, standard error; PE, phosphatidylethanolamine; Cer-NS, ceramide non-hydroxyfatty acid-sphingosine; DAG, diacylglycerol; PC, phosphatidylcholine.**

## Discussion

Hypotheses about POD development include “neuroinflammatory,” “neuronal aging,” “oxidative stress,” “neurotransmitter deficiency,” and “network disconnectivity” (Maldonado, 2013). Previous studies have found that aging, preoperative cognitive impairment, and dementia are the most important factors associated with POD. Our metabolomics results suggest that neuroinflammation, oxidative stress, energy metabolism disorders, and neurotransmitter imbalances caused by hypoxia and mitochondrial dysfunction at baseline may contribute to the risk of POD. Our current findings are not only consistent with previous pathophysiological models of delirium, but also extend these findings.

Hypoxia impairs normal function of the blood–brain barrier and promotes neurodegeneration. The storage of adenosine triphosphate (ATP) in brain tissue is very limited. If an insufficient supply of ATP is produced as a result of hypoxia or an insufficient oxygen supply, glycolysis can be promoted to compensate for this deficiency. The first step of glycolysis is the formation of glucose-6-phosphate, which is catalyzed by hexokinase. In this study, we found that the key intermediate product of glycolysis (the main pathway of carbohydrate metabolism), glucose-6-phosphate, was significantly increased in the CSF of POD patients compared with Non-POD patients (FC POD/Non-POD = 2.94, *p* = 8.85E-03), indicating that the brain tissue of POD patients might exist in a hypoxic environment. In addition, our results suggest that hypoxanthine, which is involved in the purine metabolic pathway, was decreased in the CSF of POD patients compared with Non-POD patients (FC POD/Non-POD = 0.67, *p* = 0.01). Moreover, disturbances in nucleic acid metabolism were observed. Levels of inosine in preoperative CSF also exhibited a decreasing trend in POD patients (FC POD/Non-POD = 0.82, *p* = 0.06), partially reflecting the enhanced oxidative damage to cell nuclei and mitochondrial DNA.

When ATP is exhausted, creatine can be rapidly metabolized to provide energy for nerve cells. Because insufficient ATP was produced by glycolysis in the hypoxic environment of POD patients, creatine was significantly consumed (FC POD/Non-POD = 0.75, *p* = 9.51E-03) to supplement ATP production. Deficiency of energy metabolism could induce cerebral dysfunction, as well as the associated cognitive and behavioral symptoms of delirium. Decreased oxygenation causes a failure in oxidative metabolism, which may be one cause of the problems observed in delirium, namely oxidative stress, which causes a failure of the ATPase pump system. When the pump fails, ionic gradients cannot be maintained, leading to significant influxes of sodium (Na^+^) followed by calcium (Ca^2+^), whereas potassium (K^+^) moves out of the cell (Siesjo, 1984; Kirsch et al., 1989). The influx of Ca^2+^ during hypoxic conditions is associated with a dramatic release of several neurotransmitters, particularly dopamine (DA) and glutamate (Glu). Neurotransmitter disturbances may be primary contributors to POD development. It is commonly accepted that neurotransmitter imbalances in the brain are the final common pathway in the occurrence of delirium. Thus, the pathogenesis of POD might be associated with alterations in neurotransmitter synthesis, function, and/or availability that mediate complex behavioral and cognitive changes. The most commonly described neurotransmitter changes associated with delirium include excess DA and/or Glu release.

DA, an endogenous central neurotransmitter, is the immediate precursor of norepinephrine in the catecholamine synthesis pathway. Excess DA is among the most commonly described neurotransmitter imbalances in the pathogenesis of delirium (Maldonado, 2013). Increased dopaminergic transmission is usually accompanied by reduced central cholinergic transmission, which is postulated to be associated with delirium. Yilmaz et al. (2016) studied 137 patients undergoing coronary artery bypass surgery and found that intraoperative DA was an independent risk factor for POD. Relative risk was 2.37 (95% CI: 0.18–31.28; *p* = 0.51) for total doses over 10 mg/kg and 3.55 (95% CI: 1.16–10.89; *p* = 0.02) for total doses over 30 mg/kg, indicating that high-dose DA was more likely to cause POD. In this study, we found that the CSF DA level was significantly increased in POD patients compared with Non-POD patients (FC POD/Non-POD = 13.15, *p* = 0.04), consistent with previous studies. Under impaired oxidative conditions, significant amounts of DA are released and there is a failure of adequate DA reuptake. An influx of Ca^2+^ stimulates the activity of tyrosine hydroxylase, which converts tyrosine to 3,4-dihydroxyphenylalanine to increase DA production, and further uncouples oxidative phosphorylation in brain mitochondria (Kirsch et al., 1989). Kesby et al. (2018) found that higher dopamine metabolites were associated with psychotic features. Elevation in DA availability may lead to some of the observed neurobehavioral alterations via the direct excitatory activity effects of DA, inducing apoptosis by mechanisms independent of oxidative stress (Bagnoli et al., 2020) and potentiation of the excitotoxic effects of Glu (Belov Kirdajova et al., 2020).

Widely recognized as the primary excitatory neurotransmitter in the brain, Glu plays crucial roles in cognitive function and the development of neurodegenerative disorders. Guo et al. (2017) conducted a clinical study of preoperative serum metabolites associated with POD in elderly hip fracture patients and found that preoperative serum glutamine (Gln) was significantly decreased in POD patients (FC POD/Non-POD = 0.90, *p* = 0.02). We observed similar results in preoperative CSF. The Glu level in CSF samples of POD patients was markedly increased (FC POD/Non-POD = 4.21, *p* = 0.01), while the Gln level was significantly decreased (FC POD/Non-POD = 0.64, *p* = 0.02), indicating that Glu–Gln cycle homeostasis might be disturbed, thus giving rise to cognitive impairment in elderly patients. Normally, Glu is released into the synapse, removed by astrocytes, and subsequently converted into Gln, ending its action. However, under oxidative conditions, Glu accumulates in the extracellular space because its reuptake and metabolism in glial cells is impeded by ATPase pump failure (Herrera-Marschitz et al., 2018).

Lipids have diverse biological functions, such as cellular architecture, energy storage, and cell signaling (Shevchenko and Simons, 2010) and brain lipid homeostasis plays an important role in AD and other neurodegenerative disorders. The most abundant lipid species identified in the central nervous system are cholesterol, ceramide (Cer), sphingomyelin (SM), phosphatidylcholine (PC), glucosyl ceramides, and sulfatides (Zarrouk et al., 2018). It is well-established that Cer is involved in oxidative stress, inflammation, and/or cell death, which contribute to the development of AD. Apoptosis is stimulated by Cer though the inhibition of mitochondrial electron transport and cytochrome release (Faezi et al., 2015). High levels of Cer in the brain can promote inflammatory pathways and exert negative effects on neurons during the aging process (Cutler et al., 2004). A previous study found that cytokines increased hepatic SM synthesis to increase plasma SM levels (Memon et al., 1998); moreover, elevated levels of SM have been reported in AD brains (El Gaamouch et al., 2016). Mielke et al. (2010) conducted a longitudinal population-based study involving 100 women who were followed up in six visits over 9 years. They found that serum Cer and SM varied according to the timing of memory impairment onset and may be good pre-clinical predictors (or biomarkers) of memory impairment – a deficit observed early in AD pathogenesis. Mapstone et al. (2014) performed a targeted metabolomic and lipidomic analysis and found that PC (36:6), PC (38:0), PC (38:6), PC (38:0), PC (40:1), PC (40:2), and PC (40:6) were depleted in the plasma of the Converter group, but not in the Non-Converter group. As the suppression of PC composition is itself sufficient to result in apoptosis, lacking some types of PC can be a critical factor leading to neuronal damage (Adibhatla and Hatcher, 2002). The relationship between inflammatory markers and the lipidome has been investigated. Wallace et al. performed a lipidomic analysis to study the relationship between the lipidome and inflammatory markers, and found strong negative correlations between inflammatory markers (C-reactive protein and tumor necrosis factor α) and lipids (PE and PC classes), whereas interleukin 8 had positive correlations with lipids of Cer and SM classes (Wallace et al., 2014). In our study, we found similar results indicating that preoperative CSF levels of PE and PC classes, including PE (40:6), PE (38:7e), PE (40:7e), PC (40:6), and PC (33:1), were significantly decreased in the POD group compared with the Non-POD group. However, in POD patients, preoperative CSF levels of Cer-NS and SM classes were significantly increased (*P* < 0.05). These results indicate that levels of inflammatory markers may be increased in the preoperative CSF of POD patients, and neuroinflammation plays an important part in the progression of POD.

In conclusion, we revealed the presence of alterations in multiple metabolic pathways in the POD group before surgery, including neuroinflammation, oxidative stress, and energy metabolism, which involve interactions between hypoxia and mitochondrial dysfunction, as well as neurotransmitter imbalances. These metabolic abnormalities potentially increase the fragility of the brain and contribute to POD.

## Limitations

Our study has some limitations. First, power calculations were not performed. Instead, we aimed to recruit the maximum number of patients available. We only recruited 10 POD patients and 30 Non-POD patients in our metabolomic and lipidomic analysis. Thus, further validation analysis needs to be performed in larger cohorts in future studies. Second, this work could be improved by performing the multiple reaction monitoring- mass spectrometry (MRM-MS) validation on some of the metabolites and lipids between POD and Non-POD patients. Considering the ethical issue of limited CSF, we regret to say that there were not enough CSF samples, especially POD patients’ samples, for MRM-MS validation after metabolomic and lipidomic analyses. We can only do this in the future after we have obtained enough CSF. Although we did not perform the MRM-MS validation, quality control samples were prepared by pooling equal volumes of all study instances, and were analyzed between every five samples during the entire LC–MS analytical sequence. The concentration data were normalized to sample median and take the logarithm. The methodology at each step was as rigorous as we could. We hope that researchers can pay attention to it and provide reference for more rigorous clinical design in the future.

## Data Availability Statement

The original contributions presented in the study are included in the article/Supplementary Material, further inquiries can be directed to the corresponding author/s.

## Ethics Statement

The studies involving human participants were reviewed and approved by the Beijing Jishuitan Hospital Medical Science Research Ethics Committee (JLKS201901-04). The patients/participants provided their written informed consent to participate in this study.

## Author Contributions

YH, ZL, YZ, ML, and XG designed the study. YH, WZ, YS, TL, GW, and YY collected samples and performed clinical-related analyses. JL and LZ performed metabolomic and lipidomic experiments. YH, XW, NY, YL, XM, and DH reviewed statistical analyses. YH and JL wrote the manuscript. All authors read and approved the final manuscript.

## Conflict of Interest

The authors declare that the research was conducted in the absence of any commercial or financial relationships that could be construed as a potential conflict of interest.

## Footnotes

1 http://www.reifycs.com/AbfConverter/index.html

2 https://www.metaboanalyst.ca
