# Supplementary material for: Metabolomic and Lipidomic Profiling of Preoperative CSF in Elderly Hip Fracture Patients With Postoperative Delirium
Source: Front Aging Neurosci. 2020 Oct 22;12:570210. doi: 10.3389/fnagi.2020.570210 (PMC7642614; doi:10.3389/fnagi.2020.570210)
Supplement: Supplementary file 1 [file Table_1.docx]

**Table S1. Differentiating** **lipids between POD and Non-POD groups identified from the lipidomic data**

| Pathway | Lipids | VIP | *P* Value | FC (P/N) | Trend |
| --- | --- | --- | --- | --- | --- |
| Sphingolipid metabolism | Cer-NS d40:2; Cer-NS d18:1/22:1 | 2.96 | 4.41E-05 | 2.51 | Up |
| Glycerophospholipid metabolism | PE 40:6; PE 18:0-22:6 | 2.86 | 8.00E-04 | 0.59 | Down |
| Glycerophospholipid metabolism | PE 38:7e; PE 16:1e/22:6 | 2.76 | 1.35E-03 | 0.66 | Down |
| Glycerophospholipid metabolism | PE 40:7e; PE 18:1e/22:6 | 2.70 | 1.74E-03 | 0.43 | Down |
| Glycerolipid metabolism | DAG 44:5e; DAG 22:3e/22:2 | 2.61 | 4.52E-04 | 0.47 | Down |
| Glycerolipid metabolism | LDGCC 34:1 | 2.49 | 9.05E-04 | 0.6 | Down |
| Sphingolipid metabolism | Cer-NS d50:1; Cer-NS d22:1/28:0 | 2.39 | 1.61E-03 | 2.18 | Up |
| Sphingolipid metabolism | Cer-NS d52:1; Cer-NS d22:1/30:0 | 2.27 | 2.89E-03 | 3.00 | Up |
| Sphingolipid metabolism | Cer-NS d42:4; Cer-NS d22:3/20:1 | 2.26 | 3.03E-03 | 1.79 | Up |
| Glycerophospholipid metabolism | PC 34:3 | 2.18 | 4.47E-03 | 75.52 | Up |
| Glycerophospholipid metabolism | PC 40:6; PC 18:0-22:6 | 2.12 | 1.69E-02 | 0.51 | Down |
| Glycerophospholipid metabolism | PC 33:1; PC 16:0-17:1 | 2.06 | 2.04E-02 | 0.74 | Down |
| Glycerophospholipid metabolism | PC 32:2 | 2.02 | 8.79E-03 | 1.60 | Up |
| Sphingolipid metabolism | Sphinganine 25:0 | 2.02 | 9.11E-03 | 0.42 | Down |
| Sphingolipid metabolism | SM d34:1; SM d18:1/16:0 | 1.96 | 1.17E-02 | 4.78 | Up |
| Sphingolipid metabolism | SM d44:2; SM d21:2/23:0 | 1.92 | 1.34E-02 | 1.59 | Up |
| Sphingolipid metabolism | SM d34:2; SM d14:2/20:0 | 1.84 | 1.84E-02 | 1.26 | Up |
| Glycerophospholipid metabolism | PC 37:3 | 1.82 | 1.99E-02 | 15.33 | Up |
| Glycerophospholipid metabolism | PC 33:2 | 1.80 | 2.14E-02 | 1.65 | Up |
| Sphingolipid metabolism | SM d42:2; SM d18:1/24:1 | 1.79 | 2.18E-02 | 1.27 | Up |
| Glycerolipids metabolism | TAG 48:2; TAG 14:0-16:1-18:1 | 1.78 | 2.31E-02 | 0.80 | Down |
| Sphingolipid metabolism | SM d44:3; SM d18:1/26:2 | 1.77 | 2.36E-02 | 1.52 | Up |
| Glycerolipids metabolism | TAG 56:1; TAG 18:0-20:0-18:1 | 1.74 | 2.61E-02 | 0.70 | Down |
| Glycerophospholipids metabolism | LPC 16:1-SN1 | 1.70 | 3.06E-02 | 0.65 | Down |
| Sphingolipids metabolism | HexCer-NDS d46:1 | 1.69 | 3.08E-02 | 1.37 | Up |
| Glycerolipids metabolism | TAG 26:0; TAG 8:0-8:0-10:0 | 1.67 | 3.37E-02 | 0.63 | Down |
| Glycerophospholipid metabolism | PC 40:6 | 1.63 | 3.82E-02 | 1.51 | Up |
| Sphingolipid metabolism | SM d42:3; SM d18:2/24:1 | 1.63 | 3.87E-02 | 1.23 | Up |
| Sphingolipid metabolism | Cer-NS d39:4; Cer-NS d21:2/18:2 | 1.61 | 4.12E-02 | 1.51 | Up |
| Sphingolipid metabolism | SM t60:4 | 1.59 | 4.34E-02 | 1.27 | Up |
| Glycerophospholipid metabolism | PE 40:6 | 1.56 | 4.75E-02 | 0.73 | Down |
| Sphingolipid metabolism | SM d42:4; SM d18:1/24:3 | 1.55 | 4.92E-02 | 1.25 | Up |
| Sphingolipid metabolism | SM d40:1; SM d18:1/22:0 | 1.55 | 4.97E-02 | 1.22 | Up |

VIP, variable importance in the projection; FC, fold change; Cer-NS, ceramide non-hydroxyfatty acid-sphingosine; PE, phosphatidylethanolamine; DAG, diacylglycerol; LDGCC, lysodiacylglyceryl-3-O-carboxyhydroxymethylcholine; PC, phosphatidylcholine; SM, sphingomyelin. TAG, Triacylglycerol; LPC, Lysophophatidylcholine; HexCer-NDS, Hexosylceramide non-hydroxyfatty acid-dihydrosphingosine;
